# Long noncoding RNA: noncoding and not coded

**DOI:** 10.1038/cddiscovery.2016.104

**Published:** 2017-01-09

**Authors:** Debra Toiber, Gabriel Leprivier, Barak Rotblat

**Affiliations:** 1 Department of Life Sciences, Ben-Gurion University of the Negev, Beer Sheva, Israel; 2 Department of Pediatric Oncology, Hematology and Clinical ImmunologyHeine University, Dusseldorf, Germany

Mammalian evolution exhibits extraordinary acceleration in phenotypic complexity while displaying genetic conservation. Indeed, protein-coding genes are 90% identical between mice and human, and the question arises what might be the mechanisms by which phenotypic complexity increases while the number of proteins does not? An interesting possibility is that the increase in regulatory complexity, as reflected in a more sophisticated spatio-temporal regulation on gene expression, could generate more diverse combinations of proteins and a greater number of distinct phenotypes. Importantly, unlike protein coding genes, long noncoding RNAs (lncRNA) exhibit only 50% similarity between mice and human.^1^ Are these RNA species involved in such regulation?

In recent years, lncRNA have captured the attention of biologists. LncRNA are highly abundant in the human genome, and it has been demonstrated that they have fundamental roles in a variety of biological processes. In particular, lncRNA have been shown to participate in gene regulation by binding to proteins, such as chromatin regulators, and targeting them to particular regions in the genome.^2,3^ The interaction between lncRNA and specific proteins often depends on an explicit sequence within the lncRNA,^4–6^ and there has been a surge in new techniques to investigate lncRNA-protein interactions of late.^7^ However, in some cases, it seems that lncRNA-protein interactions are promiscuous and sequence independent.^8^ Furthermore, it has been demonstrated that lncRNA regulate genes in *cis* and their transcription *per se*, not the actual sequence, is activating the adjacent gene.^9^ Considering that there are many such lncRNA/protein coding RNA gene pairs in our genome,^10^ it is important to determine the extent and functions of protein coding gene regulation by adjacent lncRNA genes.

Two recent works^11,12^ have demonstrated that a number of lncRNAs act in *cis* to regulate the transcription of neighboring genes. By analyzing 12 lncRNA loci in mouse embryonic stem cells, Engreitz *et al.*^11^ found that knocking out the promoter of five of these lncRNAs *cis-*modulated the expression of nearby genes, which were located within 5–71 kb from the promoters. To assess whether these *cis* effects were driven by the lncRNAs sequence, by the transcription process itself, or by DNA regulatory elements present in the promoter, polyadenylation signals were inserted in the studied lncRNA genes to prevent their transcription while preserving the promoter integrity. In a number of cases, the insertion of polyadenylation signals did not impact the transcription of neighboring genes,^11^ arguing that in these cases transcription of lncRNAs is not involved in modulating nearby gene expression. These experiments thus support the notion that enhancer-like DNA regulatory elements are present in such lncRNA promoters. In other cases, termination of lncRNA transcription impacted neighboring gene transcription in *cis*,^11,12^ indicating that the transcription of some lncRNAs is required for the observed *cis* effect. However, in all cases, specific lncRNA sequences were not required to mediate such effects, as exon deletion or sequence replacement did not affect nearby gene expression.^11,12^ Moreover, while a number of lncRNAs are not conserved, the DNA regulatory elements with enhancer activity present in lncRNA promoters are highly conserved,^11^ highlighting the functional importance of these *cis*-regulatory elements.

Such *cis* gene regulatory mechanism is physiologically relevant as shown in mouse models.^12^ Anderson *et al.*^12^ demonstrated *in vivo* that the transcription of the heart development regulator *Hand2* is controlled in *cis* by the lncRNA upperhand (*Uph)*, which is located 150 bp distant on the opposite strand. Gene-targeted knockout of *Uph,* but not knockin of a heterologous DNA sequence into the *Uph* locus, blocked expression of *Hand2* in mouse heart and gave rise to a similar cardiac phenotype as *Hand2* knockout embryos. The *cis* effect is therefore dependent on *Uph* transcription but not on its sequence. *Uph* transcription was found to be required to maintain histone marks characteristic of super-enhancers, and to recruit the GATA4 transcription factor to the *Hand2* cardiac enhancer.^12^

Together, these works underscore the functional importance of lncRNA genes in fine tuning the expression of an adjacent protein-coding gene in a sequence-independent manner. The idea of sequence-independent functions of lncRNA is supported by a recent study, which showed that there is no evidence for conservation of the secondary structure of known functional lncRNA.^13^ Assuming that RNA–protein interaction is structure dependent, it is tempting to speculate that lack of structural conservation in lncRNA indicates that their major functions, from an evolutionary perspective, are related to their *cis* functions. In accordance, Kapusta and Feschotte^14^ proposed an evolutionary hierarchy by which lncRNA acquire functions; first. they function by regulating an adjacent gene in *cis*, and subsequently, they acquire *trans* functions (Figure 1). This is in line with higher sequence conservation observed in older mammalian-conserved lncRNA as compared with younger, humanoid-specific lncRNA.^15^

Analysis of human lncRNA sequences indicate that they have originated from transposable elements (TE) as 75% of human lncRNA have traces of a TE.^16^ It is therefore conceivable that, during evolution, insertion of a TE at a specific location could affect the expression of its adjacent genes, their promoters, and the local chromatin landscape. In cases where this insertion increased fitness, the TE element would be selected for. Indeed, regulation of the chromatin landscape occurs at several levels as well. For example, the ‘ripple effect’ shows that the expression of a particular gene can affect the expression of its neighbors up to 100 kb away.^17^ Together, with the fact that neighboring genes are evolutionarily linked,^18^ we argue that the genomic location of lncRNA may contribute significantly to their function and their birth.

Properties of lncRNA have important implications to how we study these genes. In the study of protein coding genes, the gene sequence provides the sequence of the protein product, the domains it harbors, its conservation, and rate of changes through evolution, all of which help elucidate gene function. However, in the case of lncRNA, it would seem that there is less functional information embedded in the sequence of the gene, and their major functions might be related to their genomic location. Therefore, one of the major challenges in the field of lncRNA is to be able to integrate the genomic and transcriptomic data to infer possible functions for lncRNA genes in light of the possibility that they might be noncoding and not coded.

## Figures and Tables

**Figure 1:**
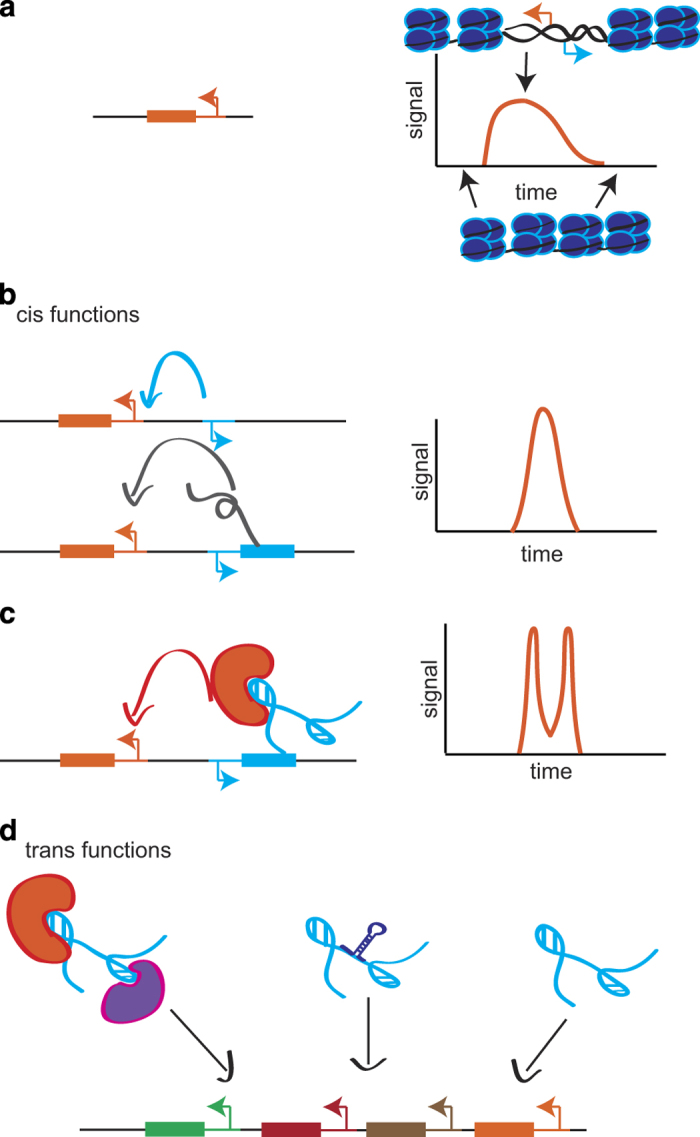
Hierarchy of lncRNA evolution linked to increase complexity of gene regulation. (**a**) the spatio-temporal regulation of the extent and strength of gene transcription is regulated by the gene’s promoter and chromatin state. (**b**) an adjacent promoter can add another layer of regulation to increase the tightness of the signal by the promoter activity directly and/or by the transcript arising for such a promoter (*cis* function). (**c**) the long noncoding transcript may bind proteins to further increase the complexity of regulation of the adjacent protein-coding gene (*cis* function). (**d**) the transcript may acquire *trans* functions such as binding to proteins and regulating their activity, sponging microRNA or binding to chromatin at distal locations.
