# Long noncoding RNA: noncoding and not coded

**DOI:** 10.1038/cddiscovery.2017.35

**Published:** 2017-08-14

**Authors:** Debra Toiber, Gabriel Leprivier, Barak Rotblat

**Correction to:** *Cell Death Discovery* (2017) **3**, 16104; doi:10.1038/cddiscovery.2016.104; published online 9 January 2017

Since the publication of this article, it has been noted that the affiliation for Gabriel Leprivier was incorrect. The correct affiliation is as follows:

^2^Clinic for Pediatric Oncology, Hematology and Clinical Immunology, Medical Faculty, Heinrich Heine University, Dusseldorf, Germany.

The authors apologise for any inconvenience caused by this error.

The original html and pdf versions of the paper have been rectified, and now carry the corrected paper.

